# The association between leisure activity and mental health in the older adults in China: amazing Guangchangwu

**DOI:** 10.3389/fpubh.2023.1291809

**Published:** 2024-01-16

**Authors:** Jiajun Yu, Ya-Ling Chiu, Sy-Ming Guu, Jying-Nan Wang

**Affiliations:** ^1^School of Innovation and Entrepreneurship, Guangzhou Huashang College, Guangzhou, China; ^2^School of Management, Guangzhou Huashang College, Guangzhou, China; ^3^College of International Business, Zhejiang Yuexiu University, Shaoxing, China; ^4^Graduate Institute of Business and Management, College of Management, Chang Gung University, Taoyuan, Taiwan; ^5^Shaoxing Key Laboratory for Smart Society Monitoring, Prevention and Control, Shaoxing, China

**Keywords:** mental health, leisure activities, older adults, Guangchangwu, in China

## Abstract

**Objective:**

Since the mental health of older adult is an important topic in the aging society, the main purpose of this study is to understand the mental health status of the older adult in China under different conditions. More importantly, although people generally believe that leisure activities can improve mental health, the impact of these activities on older adult has not yet been fully discussed. Hence, this study further explores that what kind of leisure activity is associated with mental health of the older adult given different conditions.

**Methods:**

We conducted a cross-sectional questionnaire survey to explore the relationships of various leisure activities on mental health among older adults under different demographics. This study used the Geriatric Depression Scale short forms scale (GDS-15) to assess the mental health of older adults. Based on a sample of 2,006 participants, both two-sample *t*-test and ANOVA were adopted to analyze the characteristics of mental health among specific subsamples.

**Results:**

Our findings indicated that older adults generally have higher mental health scores if they do not have chronic diseases, live with other family members, or reside in urban. First, three leisure activities including walking, Guangchangwu, and hiking have positive associations on mental health for older adults with chronic diseases. Second, the older adults living alone engaged in Guangchangwu or hiking significantly associated with their good mental health. Finally, only Guangchangwu has a significantly positive associated with the mental health of rural older adults.

**Conclusions:**

Based on our results, the government and healthcare planners can better allocate limited resources under different conditions to promote certain leisure activities, which are helpful to enhance the mental health of older adults. Guangchangwu is an activity that meets the characteristics of Chinese culture, so we further conclude that it is significantly associated with the good mental health of older adults in China.

## 1 Introduction

Population aging is a major challenge for many countries. From 2015 to 2050, the proportion of older adults in the world is estimated to nearly double, from about 12 to 22% (1). An aging society will inevitably bring many conflicts and pressures on the economic and social development such as rational allocation of social resources (2, 3), management of the pension fund (4, 5), or the older adult healthcare expenditures (6, 7). Among these issues, the mental health of the older adult is certainly not to be overlooked. Specifically, the older adult appears to reduce activity, chronic pain, weakness and more likely to experience events such as bereavement or decline in socioeconomic status after retirement (8). Therefore, older adults are more prone to mental health problems including depression, anxiety, and severe cognitive impairment. According to the WHO report, over 15% of older adult experience some type of mental health concern (1) and mental illness would lead serious economic, social, and political problems (9).

This study focuses on the issue of mental health for the older adult in China due to following two reasons. First, China is one of the fastest aging countries in the world. Statistically, the population aged over 60 exceeds 230 million, accounting for 16.7% of the total population in 2016 (10). Furthermore, the older adult population in China will expectedly reach 480 million in 2050, accounting for about 25% of the global (11). So, the issue of aging in China becomes more prominent. Second, China's medical resources are relatively inadequate compared to developed countries. For example, in 2014, the health spending in the United State, Japan, and the United Kingdom are 9,036, 4,269, and 3,989 dollars per capita, respectively. However, this number in China is only 733 dollars per capita (12). Even if the Chinese government has been committed to improving the health care system, how to maintain the mental health of the older adult under the current limited resources is an urgent issue. Therefore, the purpose of this study is first to investigate the status of the mental health for the older adult in China and then to find some ways to improve older adult's mental health.

Engagement in leisure activities undoubtedly plays a critical role in contributing to the enhancement of mental health and facilitating successful aging (13–16). Specifically, participating in leisure activities increased positive feelings and alleviated psychological problems such as loneliness and depression (17–19). In the literature, the relationship of different types of leisure activities on the mental health of the older adult has been well-supported (13, 20–22). For example, Chao (20) investigated whether mental health is affected by various leisure activities including physical activities such as walking, jogging, mountain climbing, and playing Tai-chi, and non-physical activities such as chatting, watching TV, reading the newspaper, and playing chess. These activities contributed to improving mental health, which is consistent with previous findings on leisure activities and depression studies (18, 21).

Even though there are many studies that provide evidence that the older adults participate in leisure activities to promote their mental health, to the best of our knowledge, none focuses on what kind of activities are helpful to Chinese aging society, especially the Guangchangwu. It is also known as square dancing, public dancing, plaza dancing or more vividly dancing grannies. Guangchangwu refers to “the practice of group dancing in outdoor spaces with musical accompaniment, usually in the form of a loudspeaker, and in the case of folk-style yangge Guangchangwu, with live instruments” (23). In the past decade, Guangchangwu has become a very visible cultural phenomenon in China. It is estimated that 80–100 million Chinese, mainly middle-aged and older adult, enthusiastically participate in a form of dance calisthenics called as Guangchangwu (24). It is worth noting that since the collectivist culture associated with China's agricultural society has existed for centuries, people will generate a comfortable sense of safety and belonging when following one leader with others. Therefore, this collectivistic culture makes Guangchangwu become one of the most important leisure activities for the older adults in China (23, 25). In addition to the leisure activities mentioned in the previous literature including playing chess, walking, going to the gym, hiking, we further consider China's unique Guangchangwu in this study.

In summary, the purpose of this study is to understand what types of leisure activities associated with mental health among the older adults. We address the following questions: (1) What is the mental health status of the older adult in China under different conditions? (2) What kind of leisure activities can contribute older adults related to their mental health? Combining these two questions, to explore the impact of leisure activities under various conditions, we can have a comprehensive understanding of this issue. Our findings can provide key information for healthcare planners and governments to better allocate limited resources to popularize certain leisure activities, which are helpful to enhance the older adults' mental health.

## 2 Materials and methods

### 2.1 Measurements

During the questionnaire design process, we investigated existing GDS-15 Chinese version questionnaire, types of leisure activities, and some demographic variables in our study. In addition, we also consulted two medical management professors and a specialist physician. The original questionnaire was written in English and translated into Chinese by a professor of Chinese medical management. Then two doctoral students independently translated the questionnaire into English to verify its accuracy. The original and back-translated versions were compared for conceptual equivalence and re-examined by two bilingual Chinese-English speakers. The questionnaire consisted of three sections described below.

#### 2.1.1 Older adult mental health

It is generally believed that depression a mood disorder and is the most common mental health problem among the older adult (26). Therefore, this study used the Geriatric Depression Scale short forms scale (GDS-15) to assess the mental health of the older adults. This scale was developed by Sheikh and Yesavage (27), and later translated into Chinese and validated by Mui (28). The characteristics of the GDS-15 are as follows: (1) The scale is specifically for the older adult and is currently one of the most used screening tools for depression among the older adult; (2) It includes of 15 dichotomy questions that can be answered by “Yes” or “No,” which is easy to answer and is more suitable for older people to answer questions. The total score ranges from 0 to 15, with higher scores indicate higher levels of depression. The sensitivity and specificity of the GDS-15 have been found to be high in the detection of clinical depression (29) and good reliability among the older adult in China (28, 30). A GDS score between 0 and 4 indicates that there is no presence of depression; a score of 5 and above indicates that there may be possible presence of depression (28, 30, 31). GDS higher scores indicate more severe depression, in other words, the worse the degree of mental health. Therefore, considering the presentation associated with leisure activities, we converted the GDS score to mental health, and the mental health score of 11–15 indicates is considered normal, the higher score, the less depression.

#### 2.1.2 Leisure activities

What types of leisure activities chosen by older adults were queried by the following questions: “What do you usually do during your leisure time in 2015? (Multiple choices)” Respondents were asked to choose from among five types of leisure activities including playing chess, walking, Guangchangwu, going to the gym, and hiking. In designing these leisure activities, we made reference to relevant leisure studies in China (32–34) to ensure that leisure activities are valid in the China context. Leisure activities were coded with five dummy variables.

#### 2.1.3 Demographic characteristics

Demographic variables included gender (male or female), age (in four groups: 1 = 55–59, 2 = 60–64, 3 = 65–69, or 4 = 70 and up), living status (living with family or single), living area (urban or rural), and chronic diseases (yes or no).

To identify any problems such as unclear wording or the questionnaire taking too long to execute, we conducted a pre-test in the form of a questionnaire with 71 older adult respondents before the full-scale study. According to the analysis of the results of the pre-test, we revised the questions and confirmed the final version of the questionnaire.

### 2.2 Sampling and data collection procedures

The study was a cross-sectional survey conducted by community college of Suzhou city, so the invitation letter with questionnaire were sent who seniors over 55 years old through the community activity center which is near the community college of Suzhou City. The questionnaire was freely participated in and filled out anonymously. To encourage participants to complete the questionnaire, we provided a small gift worth 10 RMB. At the recruitment, we sent out invitation letters to about 5,000 members who were 55 years or older. Totally 3,746 older adults participated in the survey from September 4 to September 12, 2015. Therefore, the response rate of this study is 74.9%. After a strict filtering and examination procedure, 1,740 participants who had given incomplete answers were excluded. Total 2,006 valid questionnaires (valid return rate = 53.6%) were left for further data analysis in the end, including 562 and 1,444 subjects for Male and female, respectively. The ethical review by committee was not operated and approved by institute during this study implemental period. All data were collected voluntarily by individuals and no further followed up by using this data.

### 2.3 Data analysis

For the continuous variables, the *t*-test (also known as Student's *T*-test) was used to exam two subsample means and examined whether if they are different from each other. In addition, the one-way analysis of variance (ANOVA) was used to determine if the variables of three or more independent groups. First, we examined whether the mental scores are significantly different in demographic characteristics by gender, chronic disease, living status, and living area. Furthermore, we demonstrated the mental health score for five leisure activities among older adults whether are different from conditions, therefore, we stratified by chronic illness (Yes = 1 vs. No = 0), living with family (Yes = 1 vs. alone = 0), and living area (Urban = 1 vs. rural = 0) to compare these two means using intendent *t*-test. The level of significance was set at a 0.05.

## 3 Results

### 3.1 Characteristics of the sample

Table 1 presents a summary of the participants' profiles. The sample is predominantly female (72%). Less than a quarter (22%) of participants are aged 55–59 years, while the other three groups accounted for ~1 quarter (26%), respectively. More than half (56%) of participants have one or more chronic diseases. In addition, the majority of participants live with other household members (89%), whereas others live alone or in a nursing home. Most of the participants live in urban and only 6% of participants live in rural areas. Table 1 also shows the subsample average of mental health score and the subsamples are determined by each demographic variable. For all samples, the average of the mental health score is 13.14. Most of the participants (89%) have good mental health (mental health score > 10) and others might have certain mental health problems (mental health score ≤ 10). Next, we want to further understand the mental health status of participants under different conditions. Therefore, we use the *t*-test or ANOVA to examine the null hypothesis, i.e., the subsample average mental health scores are equal.

**Table 1:** The demographic characteristics of participants associated with mental health (*N* = 2,006).

**Variables**	**Classification**	***N*** **(%)**		**Mean MH (SD)**	***t-*value^a^ or *F-*value^b^**	***p*-value**
**Gender**					0.043^a^	0.483
	Male	562	(28)	13.139 (2.095)		
	Female	1,444	(72)	13.143 (2.146)		
**Age (years)**					0.023^b^	0.995
	55–59	440	(22)	13.131 (1.990)		
	60–64	525	(26)	13.162 (2.179)		
	65–69	517	(26)	13.141 (2.081)		
	≥70	524	(26)	13.132 (2.250)		
**Chronic diseases**					3.234^a^	<0.001
	No	875	(44)	13.279 (2.280)		
	Yes	1,131	(56)	12.965 (1.999)		
**Living with**					1.418^a^	0.079
	Family	1,788	(89)	13.168 (2.102)		
	Alone	218	(11)	12.931 (2.352)		
**Living area**					2.371^a^	0.010
	Urban	1,894	(94)	13.175 (2.099)		
	Rural	112	(6)	12.589 (2.563)		

^a^test by T.

^b^test by F.

SD, standard deviation; MH, mental health.

Our results show that the null hypothesis is not rejected when the subsamples are determined by gender or age. However, the average mental health scores of the participants without chronic diseases are significantly higher than those with chronic diseases (*t* = 3.234, *p* < 0.001). In addition, the average mental health scores of participants who live with other family members are significantly higher than those of single or nursing home participants (*t* = 1.418, *p* < 0.10). In the end, the average mental health scores of participants in urban areas are significantly higher than those in rural areas (*t* = 2.371, *p* < 0.01). In summary, the participants generally have lower mental health scores if they have chronic diseases, live alone, or reside in rural. Therefore, we further discuss what types of leisure activities associated with mental health of participants under these three conditions.

### 3.2 Leisure activities and mental health based on with or without chronic diseases

The most frequent leisure activities were in our sample, in descending order: walking (1,577, 78.6%), Guangchangwu (844, 42.1%), hiking (383, 19.1%), play chess (243, 12.1%), and going to the gym (234, 11.7%). Table 2 reports the frequency of leisure activities and average mental health scores of participants with and without chronic disease. Our results show that participants with chronic diseases who have higher mental health scores than those who do not participate in these activities including walking (*t* = 2.428, *p* < 0.01), Guangchangwu (*t* = 3.679, *p* < 0.01), and hiking (*t* = 4.156, *p* < 0.01). But the other two types of activities, including playing chess and going to the gym, do not have significant positive associations on improving older adults' mental health.

**Table 2:** Leisure activities in participants and comparing mean scores of mental health based on chronic (*N* = 2,006).

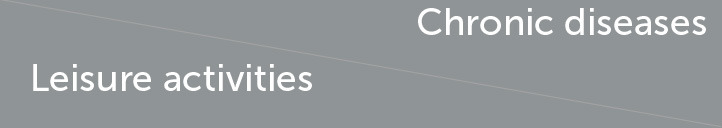	**Yes**		**No**		**Yes**	**No**
		***N*** **(%)**		***N*** **(%)**		**Mean MH (SD)**	**Mean MH (SD)**
**Playing chess**							
	Yes	127	(6)	116	(6)	13.071 (2.307)	13.509 (1.867)
	No	748	(37)	1,015	(51)	12.947 (2.277)	13.253 (2.013)
*t*-value		-		-		0.543 (ns.)	1.384^*^
*p*-value		-		-		0.287	0.084
**Walking**							
	Yes	704	(35)	873	(43)	13.061 (2.236)	13.298 (1.931)
	No	171	(9)	258	(13)	12.567 (2.420)	13.217 (2.218)
*t*-value		-		-		2.428^***^	0.529 (ns.)
*p*-value		-		-		0.008	0.299
**Guangchangwu**							
	Yes	369	(18)	475	(24)	13.285 (1.960)	13.562 (1.719)
	No	506	(25)	656	(33)	12.731 (2.464)	13.075 (2.158)
*t*-value		-		-		3.697^***^	4.223^***^
*p*-value		-		-		<0.001	<0.001
**Going to the gym**							
	Yes	98	(5)	136	(7)	13.020 (2.329)	13.294 (2.037)
	No	777	(39)	995	(49)	12.958 (2.275)	13.277 (1.995)
*t*-value		-		-		0.253 (ns.)	0.090 (ns.)
*p*-value		-		-		0.401	0.462
**Hiking**							
	Yes	150	(7)	233	(12)	13.553 (1.801)	13.648 (1.701)
	No	725	(36)	898	(45)	12.843 (2.350)	13.184 (2.060)
*t*-value		-		-		4.156^***^	3.557^***^
*p*-value		-		-		<0.001	<0.001

^*^ and ^***^ denote coefficient significance at 10, 5, and 1%, respectively.

SD, standard deviation; MH, mental health.

### 3.3 Leisure activities and mental health based on living with family or alone

Table 3 shows the average mental health scores of participants who live with their family or live alone in different leisure activities. For participants living alone, if they engage in Guangchangwu or hiking, their mental health scores are significantly higher than those who do not engage in these activities (*t* = 1.510, *p* < 0.1; *t* = 2.313, *p* < 0.05, respectively). Similarly, participants who live with their family are also involved in these two types of leisure activities and their mental health scores are also significantly higher (*t* = 5.315, *p* < 0.01; *t* = 5.050, *p* < 0.01, respectively). In addition, for the participants live with family, the mental health scores of participants with walking habits are significantly higher than those without walking habits (*t* = 2.227, *p* < 0.05). However, it is worthwhile to note that if participants are living alone, walking activities have no significant association on improving mental health (*t* = −0.553, *p* = 0.709).

**Table 3:** Leisure activities in participants and comparing mean scores of mental health based on living with family or alone (*N* = 2,006).

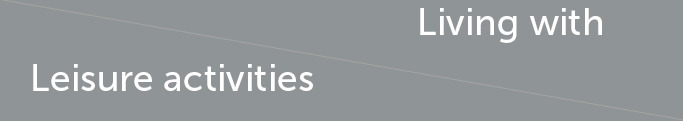	**Alone**		**Family**		**Alone**	**Family**
		***N*** **(%)**		***N*** **(%)**		**Mean MH (SD)**	**Mean MH (SD)**
**Playing chess**							
	Yes	30	(1)	213	(11)	13.000 (1.878)	13.277 (2.151)
	No	188	(9)	1,575	(79)	12.872 (2.418)	13.153 (2.096)
*t*-value		-		-		1.109 (ns.)	0.792 (ns.)
*p*-value		-		-		0.137	0.215
**Walking**							
	Yes	165	(8)	1,412	(70)	12.879 (2.300)	13.229 (2.045)
	No	53	(3)	376	(19)	13.094 (2.521)	12.939 (2.293)
*t*-value		-		-		−0.553 (ns.)	2.227^**^
*p*-value		-		-		0.709	0.013
**Guangchangwu**							
	Yes	76	(4)	768	(38)	13.237 (1.986)	13.461 (1.816)
	No	142	(7)	1,020	(51)	12.768 (2.517)	12.947 (2.270)
*t*-value		-		-		1.510^*^	5.315^***^
*p*-value		-		-		0.066	<0.001
**Going to the gym**							
	Yes	20	(1)	214	(11)	13.250 (2.337)	13.173 (2.152)
	No	198	(10)	1,574	(78)	12.899 (2.357)	13.167 (2.096)
*t*-value		-		-		0.640 (ns.)	0.037 (ns.)
*p*-value		-		-		0.263	0.485
**Hiking**							
	Yes	38	(2)	345	(17)	13.579 (1.765)	13.614 (1.729)
	No	180	(9)	1,443	(72)	12.794 (2.440)	13.061 (2.167)
*t*-value		-		-		2.313^**^	5.050^***^
*p*-value		-		-		0.012	<0.001

^*^, ^**^, and ^***^ denote coefficient significance at 10, 5, and 1%, respectively.

SD, standard deviation; MH, mental health.

### 3.4 Leisure activities and mental health based on living areas

Table 4 shows the average mental health scores of participants who live in rural or urban in different leisure activities. For the participants living in urban, if they engage in walking, Guangchangwu, or hiking, their mental health scores are significantly higher than those who do not participate in these activities (*t* = 1.677, *p* < 0.05; *t* = 4.857, *p* < 0.01; *t* = 6.227, *p* < 0.01, respectively). However, for the participants living in rural areas, only engaging in Guangchangwu has a significantly positive association on their mental health (*t* = 3.414, *p* < 0.01) and other types of leisure activities have no significant influences.

**Table 4:** Leisure activities in participants and comparing mean scores of mental health based on living area (*N* = 2,006).

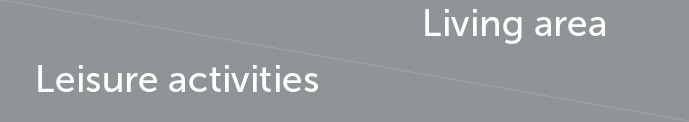	**Rural**		**City**		**Rural**	**Urban**
		***N*** **(%)**		***N*** **(%)**		**Mean MH (SD)**	**Mean MH (SD)**
**Playing chess**							
	Yes	11	(1)	232	(11)	12.727 (2.649)	13.306 (2.090)
	No	101	(5)	1,662	(83)	12.574 (2.567)	13.156 (2.101)
*t*-value		-		-		0.182 (ns.)	1.021 (ns.)
*p*-value		-		-		0.429	0.154
**Walking**							
	Yes	84	(4)	1,493	(75)	12.714 (2.398)	13.219 (2.053)
	No	28	(1)	401	(20)	12.214 (3.023)	13.010 (2.258)
*t*-value		-		-		0.796 (ns.)	1.677^**^
*p*-value		-		-		0.216	0.047
**Guangchangwu**							
	Yes	49	(2)	795	(40)	13.469 (2.237)	13.439 (1.806)
	No	63	(3)	1,099	(55)	11.905 (2.607)	12.984 (2.270)
*t*-value		-		-		3.414^***^	4.857^***^
*p*-value		-		-		<0.001	<0.001
**Going to the gym**							
	Yes	9	(1)	225	(11)	12.667 (3.279)	13.200 (2.115)
	No	103	(5)	1,669	(83)	12.583 (2.511)	13.171 (2.098)
*t*-value		-		-		0.075 (ns.)	0.191 (ns.)
*p*-value		-		-		0.471	0.424
**Hiking**							
	Yes	26	(1)	357	(18)	12.462 (2.565)	13.695 (1.637)
	No	86	(4)	1,537	(77)	12.628 (2.576)	13.054 (2.176)
*t*-value		-		-		−0.290 (ns.)	6.227^***^
*p*-value		-		-		0.613	<0.001

^**^ and ^***^ denote coefficient significance at 10, 5, and 1%, respectively.

SD, standard deviation; MH, mental health.

## 4 Discussion

Since older adults' mental illness may cause serious economic, social, and political problems, it is worth to pay more attention to this topic. The aim of this study is to investigate the mental health status under different demographic variables and to explore the relationship between leisure activities and older adults' mental health in China. Our empirical results indicate that, under three different conditions, there is a significant difference in the mental health of the older adults. First, mental health is usually in a reciprocal relationship with chronic disease (35). In other words, when an older adult suffers from a chronic disease, his/her mental health will be worse. Second, lack of social support could seriously affect the loneliness and wellbeing of the older adults in their later years. Chen and Feeley (8) indicate that spouses and children have a positive influence in fulfilling intimate aspects of social interaction. Thus, older adults lived with other household members usually receive more support from their families, which will result in better mental health. Third, past research has pointed out that for most people, the state of mental health in the rural is better than in urban areas (36). However, our results show that the mental health of the rural older adults is relatively worse, that may be due to rural pressures and economic difficulties (37, 38). In summary, we conclude that the older adults without chronic diseases (35), lived with other household members (39), and resided in urban (40), have better mental health.

Previous studies have revealed that the older adults engage in certain types of leisure activities can improve their psychological wellbeing (13, 18, 20). Similarly, our results also support the fact that participating in leisure activities usually contribute to the mental health of older adults. According to previous conclusions, the older adults without chronic diseases (35), lived with other household members (39), and resided in urban (40), have better mental health. Then, we further explore the relationship between specific types of leisure activities and mental health of the older adult in these three different situations. There are three interesting findings in this study. First, walking, Guangchangwu, and hiking are associated with the mental health of older adults who have certain types of chronic diseases. Nevertheless, there is no evidence to support that playing chess or going to the gym has a significantly positive influence on the mental health of older adults under the same condition. Therefore, the older adults with chronic illnesses are more likely to make their mental health better by participating in outdoor activities.

Second, unlike outdoor activities that help improve chronic conditions, these older adults living alone may want to be accompanied by others. It should be noted that the cultural tradition in China especially emphasizes the family system and collectivism, that implies family is usually a major mental support (41). Older adults living alone may seek similar mental support by contacting with others, but leisure activities such as walking do not require contact with the crowd. Therefore, for older adults living alone, Guangchangwu or hiking can contribute to facilitating their mental health, but other activities do not induce any significant associations. Finally, in addition to Guangchangwu, the other four leisure activities have no significant influence on the mental health of the rural older adults. It is interesting that hiking in the first two conditions is associated with mental health of the older adults, but it does not have the same association on rural older people. We speculate that the possible reason is that the daily activities of rural older adults have always been in the mountains or fields, so hiking may not be regarded as a leisure activity for them. In other words, hiking is a little boring for rural older adults, but dancing may be more fun. Thus, in our study, Guangchangwu is the most effective in promoting the mental health of the older adults in the rural.

## 5 Conclusions

In summary, we conclude that Guangchangwu is a universal solution for improving mental health. For older adults with the disease, since the outdoor exercise attribute can enhance physical health, better physical health usually comes with better mental health; for older adults living alone, the social support attribute makes they are easily touching in others; for rural older adults, the funny attribute may let them more willing to join this activity, so that they might tend to participate physical and mental health and receive more social supports. Based on these findings, we can provide some implications for the government or medical institution. Since the older adults with chronic diseases, living alone or living in rural areas generally have poor mental health, the government or care institutions must pay more attention to the older adults in these three situations. In addition, it is well-known that leisure activities generally can improve mental health, but advocating which type of leisure activities should depend on the situations of older adults. Government institutions should allocate limited resources effectively to provide useful support for different older adults. For example, there is no need to encourage the rural older adults to hike or climb, because they may have done this every day. It is worth mentioning that Guangchangwu has attracted the attention of the Chinese people and government because of its increasing popularity and ubiquity in public spaces. Guangchangwu brings vitality and uplifting energy that play an important role in providing social support, which has been recommended as an intervention strategy to help older adults on mental health. Therefore, our results found that Guangchangwu is a good solution to promote the mental health of older adults in China. In the practical view, the government can make out more applicable policies and places for Guangchangwu. In this way, older adults are willing to do more outdoor exercise, close contact with more people, and reduce their loneliness.

## 6 Limitations and directions for future research

Guangchangwu has benefits for older adult in improving cognitive function (42), memory (43), skeletal health (44), and life satisfaction (45). There are some limitations in this study. First, because our sample is from an older adult community college in Suzhou, there may be some bias, for example, these older adults may have better physical and mental health. In addition, since Guangchangwu may be a manifestation of the Chinese collectivist culture (25), social customs in different regions will certainly affect its effectiveness in improving mental health. In other words, the results are not a universal reflection of the older adults in China and limit the generalization of our findings. Therefore, future research needs to be conducted with more diverse samples, such as older adults in other provinces or ethnicities, to better understand the correlation between various leisure activities and mental health of the older adults. Second, to make the questionnaire easier to be answered by older adults, we only used dichotomy questions to ask whether they had participated in a certain leisure activity but ignored the degree of participation. Specifically, participating in leisure activities every day or once a week may have different associations on mental health. It may be helpful to adopt additional questions that cover the broader concept of participation in leisure activities. For example, we can ask questions such as “How often do you participate in this leisure activity per week?” or “How many minutes do you engage in this leisure activity each time?” These questions allow us to measure the degree of participation for older adults in various leisure activities so that we can further explore whether increasing leisure time is associated with the mental health of older adults. Finally, the cross-sectional research design used only reflects the status of the survey participants at one specific second. Thus, this research design restricts us to conduct the causal inference. Specifically, we cannot be sure that participating in certain leisure activity is the cause of a higher mental health of the older adults. For example, more optimistic older adults are more willing to participate in leisure activities. In order to determine the causal relationship between leisure participation and mental health, further studies can establish a longitudinal research design to provide more information about the development of older adults' behaviors (46).

## Data availability statement

The datasets presented in this study can be found in online repositories. The names of the repository/repositories and accession number(s) can be found in the article/supplementary material.

## Ethics statement

Ethical approval was not required for the studies involving humans because the questionnaire was filled out voluntarily and anonymously. All data were collected voluntarily by individuals and no further followed up by using this data. The studies were conducted in accordance with the local legislation and institutional requirements. Written informed consent for participation was not required from the participants or the participants' legal guardians/next of kin in accordance with the national legislation and institutional requirements because the ethical review by committee was not operated and approved by institute during this study implemental period.

## Author contributions

JY: Conceptualization, Data curation, Supervision, Writing—original draft, Writing—review & editing. Y-LC: Data curation, Formal analysis, Methodology, Writing—original draft. S-MG: Conceptualization, Formal analysis, Supervision, Writing—review & editing. J-NW: Methodology, Writing—original draft.
